# Does Generative Artificial Intelligence Improve Students’ Higher-Order Thinking? A Meta-Analysis Based on 29 Experiments and Quasi-Experiments

**DOI:** 10.3390/jintelligence13120160

**Published:** 2025-12-05

**Authors:** Yan Zhao, Yuhe Yue, Zhonghua Sun, Qiang Jiang, Gangsheng Li

**Affiliations:** 1 School of Education, Changchun Normal University, Changchun 130032, China; zhaoyan@ccsfu.edu.cn (Y.Z.); qz202401074@stu.ccsfu.edu.cn (Y.Y.); 2 School of Information Science and Technology, Northeast Normal University, Changchun 130017, China; jiangqiang@nenu.edu.cn; 3 Department of Education, Ocean University of China, Qingdao 266100, China

**Keywords:** Generative Artificial Intelligence, higher-order thinking, critical thinking, creativity, problem-solving ability, meta-analysis

## Abstract

The widespread application of Generative Artificial Intelligence (Gen-AI) is transforming educational practices and driving pedagogical innovation. While cultivating higher-order thinking (HOT) represents a central educational goal, its achievement remains an ongoing challenge. Current evidence regarding the impact of Gen-AI on HOT is relatively fragmented, lacking systematic integration, particularly in the analysis of moderating variables. To address this gap, a meta-analysis approach was employed, integrating data from 29 experimental and quasi-experimental studies to quantitatively assess the overall impact of Gen-AI on learners’ HOT and to examine potential moderating factors. The analysis revealed that Gen-AI exerts a moderate positive effect on HOT, with the most significant improvement observed in problem-solving abilities, followed by critical thinking, while its effect on creativity is relatively limited. Moderation analyses further indicated that the impact of Gen-AI is significantly influenced by experimental duration and learners’ self-regulated learning (SRL) abilities: effects were strongest when interventions lasted 8–16 weeks, and learners with higher SRL capacities benefited more substantially. Based on the research findings, this study proposed that Gen-AI should be systematically integrated as a targeted instructional tool to foster HOT. Medium- to long-term interventions (8–16 weeks) are recommended to enhance learners’ problem-solving and critical thinking abilities. At the same time, effective approaches should also be explored to promote creative thinking through Gen-AI within existing pedagogical frameworks. Furthermore, individual learner differences should be accounted for by adopting dynamic and personalized scaffolding strategies to foster SRL, thereby maximizing the educational potential of Gen-AI in cultivating innovative talents.

## 1. Introduction

Higher-Order Thinking (HOT), as a core competency of 21st-century skills, has been internationally recognized as a central objective for promoting students’ holistic development and fostering innovative capabilities. The Organization for Economic Co-operation and Development (OECD 2019) emphasizes that in an increasingly complex social environment, higher-order thinking skills, such as critical thinking and creativity, hold profound educational value and societal significance. Several countries, including the United States and Singapore, have formally incorporated HOT into their national education policy frameworks, establishing it as a strategic competency essential for future learners (OECD 2019; Ministry of Education Singapore 2021).

In recent years, the rapid development of artificial intelligence (AI), particularly Generative Artificial Intelligence (Gen-AI), has ushered HOT cultivation into a new stage with disruptive potential. Since the launch of ChatGPT in late 2022, Gen-AI has profoundly reshaped the interactions between humans, knowledge, and technology through its advanced natural language processing and multimodal generation capabilities (Arista et al. 2023; Costa et al. 2024). This technology can instantaneously generate diverse learning resources, including text, images, and videos, significantly enhancing the accessibility of educational content and the personalization of learning support. Such advancements not only expand learners’ cognitive exploration possibilities but also drive a shift in educational paradigms from traditional knowledge transmission toward technology-enhanced deep learning.

Empirical evidence suggests that Gen-AI demonstrates considerable potential in fostering HOT. By providing rich, contextualized learning materials, Gen-AI can support the development of critical thinking and facilitate multi-path problem solving (Li et al. 2024b; Pujawan et al. 2022). Furthermore, its real-time feedback mechanisms stimulate creative thinking and encourage the exploration of diverse solutions. Leveraging highly interactive and dynamic support, Gen-AI can assist learners in engaging with more complex, higher-order cognitive processes, thereby effectively promoting the systematic development of HOT (Huang et al. 2024a).

However, the integration of Gen-AI in education also raises concerns. Studies indicate that excessive reliance on AI-generated content may weaken learners’ self-directed learning and self-regulation capabilities (Sun et al. 2025). Biases and factual inaccuracies embedded within algorithms may mislead judgment and hinder the development of critical thinking. Scholars further caution that inappropriate use of Gen-AI could constrain autonomous exploration, suppress creativity, and introduce ethical, privacy, and academic integrity risks (Costa et al. 2024). These issues underscore the urgency of carefully examining the mechanisms and boundaries of Gen-AI’s impact on HOT.

Overall, despite preliminary progress in research on the relationship between Gen-AI and HOT, existing findings remain fragmented and inconsistent. Some studies highlighted the positive effects of AI technologies on enhancing critical thinking, creativity, and problem-solving skills, while others warn of potential cognitive dependency and associated risks. Most existing research is limited to small-scale experiments or case studies, lacking cross-disciplinary, multi-contextual integration, with insufficient attention to underlying mechanisms and moderating variables. Therefore, it is imperative to employ the meta-analysis method to synthesize existing empirical evidence quantitatively, systematically assess the overall effects of Gen-AI on HOT, and explore its mechanisms and boundary conditions, thereby providing robust theoretical and evidence-based guidance for educational practice and policy development.

## 2. Literature Review

### 2.1. Conceptualizing HOT

The theoretical origins of HOT can be traced back to Benjamin Bloom’s (1956) taxonomy of educational objectives. Bloom’s framework categorized cognitive goals into six hierarchical levels—knowledge, comprehension, application, analysis, synthesis, and evaluation—marking the first systematic distinction between higher-order and lower-order cognitive processes. Anderson and Krathwohl (2001) later revised Bloom’s taxonomy, reorganizing cognitive processes into remembering, understanding, applying, analyzing, evaluating, and creating, and notably emphasizing “creating” as the pinnacle of cognitive development. This revision further clarified the progression from foundational cognition toward critical evaluation and innovative thinking. From an information-processing perspective, Lewis and Smith (1993) proposed that HOT constitutes a systematic cognitive process through which individuals reorganize, extend, and deeply process information, integrating it with existing knowledge structures to achieve goals or solve complex problems. Despite variations in conceptual definitions across the literature, there is a broad consensus that HOT represents an integrated cognitive capacity, highly dependent on metacognitive awareness and complex cognitive functions, characterized by more profound, more complicated, and reflective thinking processes (including classic dimensions such as analysis, evaluation, and creation/synthesis) that demonstrate both cognitive sophistication and holistic integration (Wang et al. 2019; Ilgun Dibek et al. 2024).

To elucidate the multi-dimensional structure of HOT, scholars have examined its constituent elements from diverse disciplinary perspectives. Discipline-specific definitions have emerged as a primary research approach. Table 1 summarizes the core dimensional classifications of HOT in representative studies across disciplines.

Although the specific classifications of HOT vary across disciplinary contexts, there is widespread agreement that its core components comprise critical thinking, creative thinking, and problem-solving ability-a consensus supported by cross-disciplinary studies (Yang 2015; Hwang et al. 2018; Alkhatib 2022). These three dimensions manifest distinct domain-specific characteristics and are the most frequently activated cognitive processes in twenty-first-century skills. King et al. (1998) early on highlighted that HOT is not isolated cognitive behavior but a dynamic, coordinated system of cognitive skills. When confronted with complex problem-solving situations, these skills are activated synergistically: critical thinking enables the analysis and evaluation of information and rapid identification of problem essence (Li et al. 2024b; Liu et al. 2024a); creative thinking facilitates the exploration of novel approaches and generation of diverse solutions (Li et al. 2024b; Pujawan et al. 2022); and problem-solving ability is responsible for constructing and implementing effective strategies (Li et al. 2024b; Adijaya et al. 2023). The integrated and contextually adaptive nature of these multidimensional capacities constitutes a defining feature that distinguishes HOT from lower-order cognitive processes.

### 2.2. The Positive Impact of Gen-AI on the Cultivation of HOT

Currently, research on the relationship between Gen-AI and students’ HOT development presents a diversity of theoretical perspectives. Although preliminary empirical studies have highlighted the potential value of Gen-AI in educational applications, significant debate remains regarding its functional positioning and actual effectiveness within instructional systems.

Empirical evidence provides strong support for these claims. Costa et al. (2024) found that students who completed integrative learning tasks with the assistance of ChatGPT demonstrated significant improvements in higher-order cognitive abilities, particularly in text comprehension and critical thinking. In the context of interdisciplinary instruction, Lee et al. (2024) developed and validated a Gen-AI-based “Guided Chemistry Learning Assistant” (GCLA). By simulating expert-guided strategies, this tool effectively enhanced students’ critical thinking, multi-step problem-solving skills, and innovative application in chemistry. Furthermore, Li et al. (2024b) conducted experimental research using InquiryGPT, providing additional evidence that Gen-AI can facilitate HOT development. The study demonstrated that the tool, through a structured inquiry-based learning framework, effectively strengthened students’ systematic reasoning and metacognitive skills, offering empirical support for the role of Gen-AI in promoting HOT.

From the perspectives of cognitive and learning sciences, scholars have systematically examined the mechanisms through which Gen-AI influences the core dimensions of HOT—namely critical thinking, creativity, and problem-solving ability-focusing on its instructional applications and cognitive empowerment pathways.

#### 2.2.1. Critical Thinking

Research indicates that Gen-AI, leveraging its high interactivity and scaffolded learning features, can effectively foster students’ critical thinking. Guo et al. (2023) found that embedding AI chatbots in high school debate activities significantly enhanced students’ critical thinking, particularly in argument construction and evidence evaluation. Zheng et al. (2024) further reported that in collaborative learning environments, students strengthened their evaluative and reflective abilities by filtering and questioning AI-generated content and verifying information reliability. Bailey et al. (2021), drawing on cognitive development stage theory, emphasized that AI-generated prompts play a key role in guiding novice learners into critical reasoning processes.

#### 2.2.2. Creativity

In the domain of creativity, Gen-AI extends both learning experiences and cognitive possibilities beyond traditional instructional frameworks. Compared with teacher-led question-and-answer modes, Gen-AI requires students to construct queries to obtain targeted feedback actively, thereby cultivating initiative in knowledge exploration and divergent thinking (Alzubi et al. 2025). By providing personalized support, real-time responsiveness, and multimodal content generation, Gen-AI offers a novel technological pathway for stimulating creativity. Huang et al. (2024a) integrated AI-generated content (AIGC) into educational product design to enhance students’ HOT, demonstrating that AIGC can effectively guide learners through advanced cognitive processes such as analogical reasoning and conceptual restructuring. Moreover, by offering open-ended and heuristic feedback, Gen-AI fosters a risk-tolerant and exploratory learning environment, further promoting the development of creative thinking.

#### 2.2.3. Problem-Solving Ability

Regarding problem-solving ability, Gen-AI primarily supports students’ development of systematic problem-solving skills through mechanisms such as multi-path solution provision, response evaluation, and iterative feedback. Sun et al. (2024) found that ChatGPT not only offers alternative problem-solving strategies but also assists students in validating and optimizing solutions, significantly improving problem-solving efficiency and quality. Xu et al. (2024) introduced AI-based chatbots to facilitate gamified learning in information technology courses, demonstrating that such tools support the development of adaptive problem-solving skills in dynamic contexts. Additionally, Cam and Kiyici (2022) reported that AI-assisted learning environments in robotics programming effectively enhance students’ computational thinking and complex problem decomposition abilities, further consolidating the positive impact of Gen-AI in this domain.

### 2.3. Potential Risks and Challenges of Gen-AI in Fostering HOT

Although Gen-AI demonstrates considerable potential in fostering HOT skills, some scholars adopt a more cautious stance toward its application in education, emphasizing that without the guidance of sound pedagogical principles, its use may pose risks to students’ cognitive development. Several studies have underscored the limitations of Gen-AI’s actual effectiveness, noting that its impact is highly contingent on specific learning contexts and, in some instances, may even hinder the development of critical thinking and creativity.

Lu et al. (2024) reported that, under certain conditions, the use of Gen-AI exerted adverse effects on students’ HOT skills, suggesting that its integration does not always yield the anticipated cognitive benefits and may, in fact, impede intellectual growth. Similarly, Sun et al. (2024), in the context of programming education, observed that students often relied on ChatGPT-generated code without engaging in debugging or analysis. Such reliance not only weakened their problem-solving abilities but also reduced their depth of understanding of programming concepts. In a related vein, Putra et al. (2023) noted that students frequently accepted AI-generated content uncritically, lacking awareness of the need to question and verify its reliability and accuracy. This dependence risks further constraining the development of critical thinking and creative problem-solving abilities.

In addition, research by Kooli (2023) and Maniktala et al. (2023) has suggested that frequent reliance on Gen-AI-based dialogs may negatively impact students’ academic autonomy and capacity for knowledge internalization, fostering cognitive dependence and diminishing their ability to solve problems independently. Notably, Maniktala et al. (2023) also found that, in specific problem-solving tasks, university students who refrained from using Gen-AI outperformed their peers who did, further highlighting the dual-edged nature and potential risks of Gen-AI in educational applications. Therefore, it is imperative to strategically leverage moderating variables to mitigate these risks and ensure that Gen-AI actively contributes to the development of students’ HOT.

### 2.4. Potential Moderators

Empirical evidence indicates that the impact of Gen-AI on the development of learners’ HOT skills is highly context-dependent and conditional. Its facilitative effects are neither linear nor universally consistent but are instead constrained by multiple moderating factors. Zhou et al. (2024) identified self-regulated learning (SRL) as a critical mediating variable linking Gen-AI use to the enhancement of critical thinking and problem-solving skills. Ex-tending this line of inquiry, Xu et al. (2024) found that learners with weaker SRL capacities tend to rely more heavily on immediate feedback and external assistance, exhibiting tendencies toward unstructured inquiry and spontaneous dialog. These findings underscore the central role of individual differences in shaping Gen-AI–driven learning trajectories and outcomes, while simultaneously revealing potential educational challenges.

Beyond individual differences, temporal and group-level variations also influence how Gen-AI affects HOT development. Li et al. (2024a) and Li and Wang (2023) reported that the duration of Gen-AI use significantly conditions its impact on HOT, highlighting the importance of considering intervention length and continuity in instructional design. Similarly, Zhang and Zhu (2022) demonstrated that the effects of AI-driven conversational tools on creativity vary substantially across educational levels. Learners’ age also moderates the relationship between Gen-AI and the development of HOT. Owing to differences in cognitive structures and developmental needs across age groups, learners interact with AI in fundamentally distinct ways. These variations in interaction patterns directly shape the pathways through which Gen-AI facilitates HOT, as well as the magnitude of its eventual effects (Larson et al. 2024; Zhao et al. 2025).

In addition, instructional approaches critically shape the extent to which Gen-AI supports higher-order learning. Kimmel (2024) argued that embedding Gen-AI within project-based learning (PBL) environments can foster creative problem posing, integrative knowledge synthesis, and iterative design. Complementarily, the empirical study by Gonsalves (2024) found that in Socratic inquiry-based teaching, limiting the functions of Gen-AI to proposing counterexamples, challenging assumptions, and identifying weak evidence can significantly enhance learners’ metacognitive reflection and critical thinking skills.

Taken together, these findings suggest that the effectiveness of Gen-AI in fostering HOT is contingent upon a constellation of contextual and individual factors, underscoring the need for nuanced and context-sensitive strategies in both research and practice. At the same time, the conditional nature of its effects highlights the potential risks and challenges associated with unguided or poorly structured integration of Gen-AI into educational settings.

### 2.5. Related Reviews and Meta-Analyses on the Impact of Gen- AI on HOT

To date, several scholars have employed meta-analytic approaches to examine the impact of Gen-AI on learners, with most studies focusing on outcomes such as academic performance, learning engagement, and learning development (Deng et al. 2025; Wang and Fan 2025; Heung and Chiu 2025). Although HOT is occasionally mentioned in these discussions, the outcome variables are not differentiated based on cognitive levels, nor are HOT skills specifically assessed.

Furthermore, although several literature reviews have attempted to synthesize evidence on the effectiveness of AI in educational contexts (e.g., Zawacki-Richter et al. 2019), recent meta-analyses (García-Martínez et al. 2023) in this domain exhibit notable limitations in their scope and coverage. Most of the literature reviewed was collected up to the end of 2024. For instance, Zheng et al. (2023) consolidated research from the past two decades on the impact of AI technologies on learning outcomes and learner cognition, while Hwang (2022) focused specifically on the effects of AI agents on elementary students’ mathematics achievement. Although Ilgun Dibek et al. (2024) systematically reviewed the influence of AI tools on HOT, their analysis was limited to literature published up to 2023, emphasized general AI tools, and did not specifically incorporate studies related to Gen-AI. Collectively, these studies fail to capture the most recent empirical evidence emerging from the rapid advancements in Gen-AI.

As a core cognitive competency of the twenty-first century, HOT warrants more dedicated and in-depth research attention, particularly in the context of Gen-AI’s increasing integration into educational environments. Therefore, a systematic review and synthesis of existing empirical studies on the impact of Gen-AI on learners’ HOT is both timely and necessary. Such research not only helps to distinguish HOT from other learning outcomes clearly but also elucidates the underlying mechanisms through which Gen-AI influences HOT and identifies potential moderating factors, thereby providing theoretical grounding for the effective and targeted application of Gen-AI in education.

### 2.6. The Present Study

In light of the inconsistencies observed in prior research, this study employs a meta-analysis approach, synthesizing data from 59 experiments and quasi-experiments reported across 29 sources. The primary objective is to ascertain both the direction and magnitude of Gen-AI’s impact on students’ HOT. Guided by this objective, the meta-analysis seeks to address the following core research questions:

Q1. What is the overall effect size of Gen-AI on students’ HOT? Separately, what are the effect sizes of Gen-AI on the three levels of HOT (critical thinking, creativity, and problem-solving skills)?
Q2. Do moderating variables—such as intervention duration, educational level, instructional method, and self-regulated learning ability—impact the relationship between Gen-AI and the development of HOT? If so, how do they moderate the effect of Gen-AI on students’ HOT?

## 3. Methods

This study employs a meta-analysis approach to systematically synthesize existing empirical research on the impact of Gen-AI on students’ HOT. Further analyses were carried out using Comprehensive Meta-Analysis (CMA) 3.7. This study adopts Hedges’s g as the effect size measure. The interpretation of effect sizes follows Cohen’s (1992) guidelines: values below 0.2 indicate a small effect, values between 0.2 and 0.5 represent a moderate effect, values between 0.5 and 0.8 suggest a significant effect, and values exceeding 0.8 denote a largely significant effect.

### 3.1. Data Sources and Search Strategies

To ensure a comprehensive and systematic collection of relevant research, this study conducted a structured literature search across multiple academic databases, including Web of Science, IEEE Xplore, ScienceDirect, Springer, Google Scholar, China Online Journals, and China National Knowledge Infrastructure (CNKI). The search terms included generative artificial intelligence, Gen-AI, ChatGPT, chatbot, higher-order thinking, HOT skills, creativity, creative thinking, critical thinking, problem solving, and problem-solving ability. Boolean operators “OR” and “AND” were used to refine the search, allowing for broad keyword matching and citation tracking. Additionally, a snowballing search strategy was employed, wherein references cited in the selected papers were further examined to enrich the dataset. Given that Gen-AI had not been widely applied before the release of ChatGPT in November 2022, this study includes only literature published after this date, in which the search ended in August 2025.

### 3.2. Eligibility Criteria

As a result, 400 relevant studies were initially identified. However, not all retrieved studies met the eligibility criteria for meta-analysis. Except for excluding gray literature, the following selection criteria were applied: (1) Repetitive articles should be excluded. (2) The research topic must analyze the correlation between Gen-AI and HOT (e.g., critical thinking, creativity, problem-solving). (3) The research method must be experimental or quasi-experimental. (4) It must possess sufficient quantitative data for statistical analysis, such as data like means and standard deviations. (5) The articles must be publicly published or accessible. (6) The articles must be in English or Chinese.

**Figure 1:**
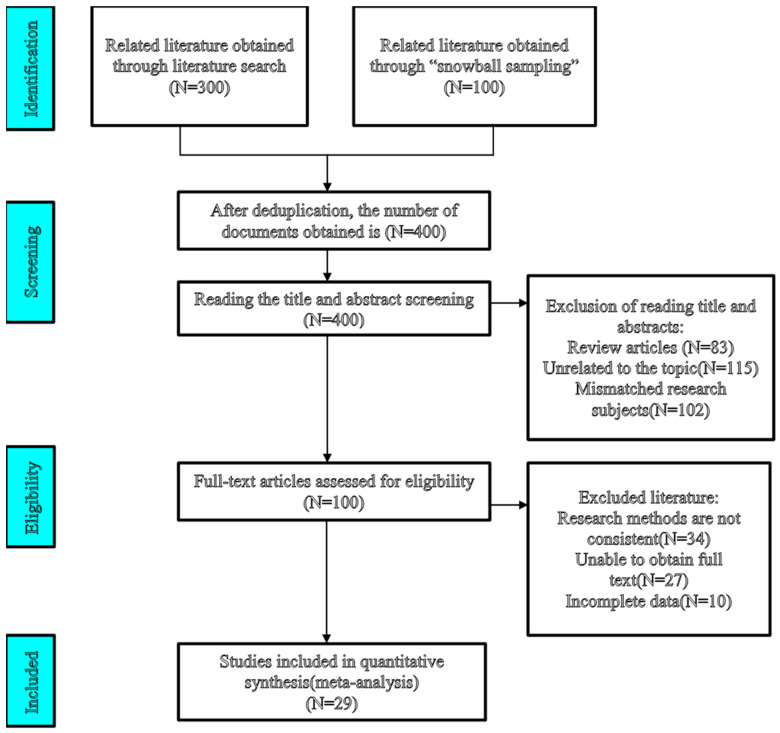
Study search and selection process.

Following these selection criteria, a total of 29 studies, comprising 59 effect sizes, met the inclusion criteria and were retained for subsequent meta-analysis. The process of literature screening is illustrated in Figure 1.

### 3.3. Literature Coding

To ensure coding reliability, two researchers independently conducted the coding process. First, the primary author performed the initial coding, followed by a secondary researcher who reviewed and validated the coded data, documenting the review date. Inter-coder reliability was assessed using Cohen’s k, which yielded a value of 0.84, indicating excellent agreement beyond chance. Any discrepancies in coding were resolved through discussion until consensus was reached. The coding framework included the following dimensions: Descriptive information (author(s), and year of publication); Quantitative data (sample size, mean values, and standard deviations); Assessment dimensions (creativity, critical thinking, and problem-solving skills), and Moderator variables. The detailed coding specifications for the moderator variables are presented in Table 2.

### 3.4. Publication Bias Test

Publication bias refers to the tendency for published studies to be preferentially included in literature reviews or meta-analyses, while unpublished studies—often with null or non-significant results—are overlooked. This can inflate the overall effect size and compromise the validity of the meta-analytic conclusions. To assess the presence of publication bias in the current study, we employed both funnel plot analysis and Egger’s regression test (Peters et al. 2008; Van der Willik et al. 2021).

**Figure 2:**
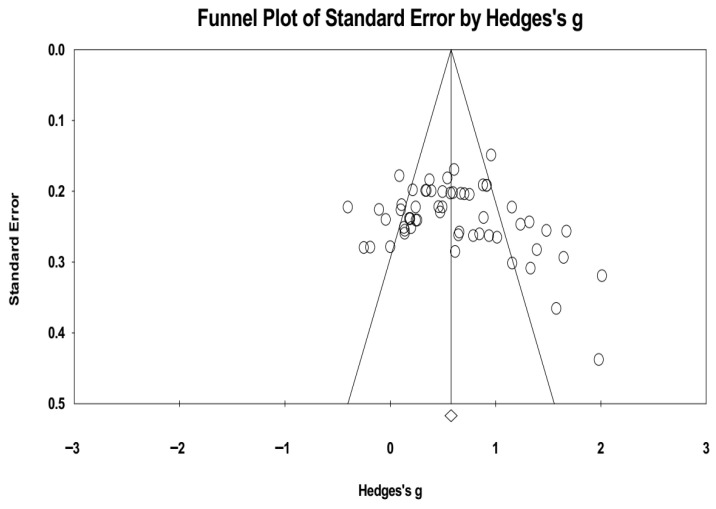
Funnel plot of standard error by Hedges’s g.

The assessment of publication bias revealed no strong evidence of severe distortion in the current meta-analysis. The funnel plot constructed from the 29 included studies showed that a total of 8 individual data points fell outside the slope lines, but a generally symmetrical distribution of data points around the mean effect size was observed (see Figure 2). However, visual inspection suggested a possible slight skew to the right, which corresponds with the findings of Egger’s test.

To corroborate this visual assessment, Egger’s regression test was performed. The test produced a *t*-value of 1.871 with a corresponding *p*-value of 0.066, which approached but did not reach the threshold for statistical significance (t < 1.96, *p* > 0.05). This finding could indicate mild publication bias. Taken together, these results indicate that the meta-analytic findings are unlikely to be significantly affected by publication bias, thereby enhancing the robustness and credibility of the study’s conclusions.

### 3.5. Heterogeneity Test

In meta-analyses, heterogeneity may arise due to differences in study design, sample characteristics, and measurement approaches among the included studies. Such variability in effect sizes can result from random sampling errors or systematic differences across study groups. Therefore, it is essential to assess and quantify heterogeneity using appropriate statistical methods.

In the present study, heterogeneity was evaluated using Cochran’s Q test and the I^2^ statistic. Cochran’s Q test is used to determine whether the variability in effect sizes across studies is greater than would be expected by chance. Conventionally, a non-significant Q value (*p* > 0.1) indicates low heterogeneity, suggesting that a fixed-effects model may be appropriate, whereas a significant Q value (*p* < 0.1) suggests that heterogeneity is present and that a random-effects model should be considered (Zhang and Hu 2019). To further quantify the extent of heterogeneity, the I² statistic is used. It ranges from 0% to 100%, with values below 25% typically regarded as low, between 25% and 50% as moderate, and above 75% as high (Higgins et al. 2003).

The analysis revealed significant heterogeneity among the included studies, as indicated by the Q statistic (Q = 255.208, *p* < 0.001; see Table 3). The I^2^ value was 77.273%, suggesting a moderate level of heterogeneity across studies. Accordingly, a random-effects model was employed to yield a more robust and generalizable estimation of the overall effect size.

## 4. Results

### 4.1. The Analysis of the Overall Effect Size

**Figure 3:**
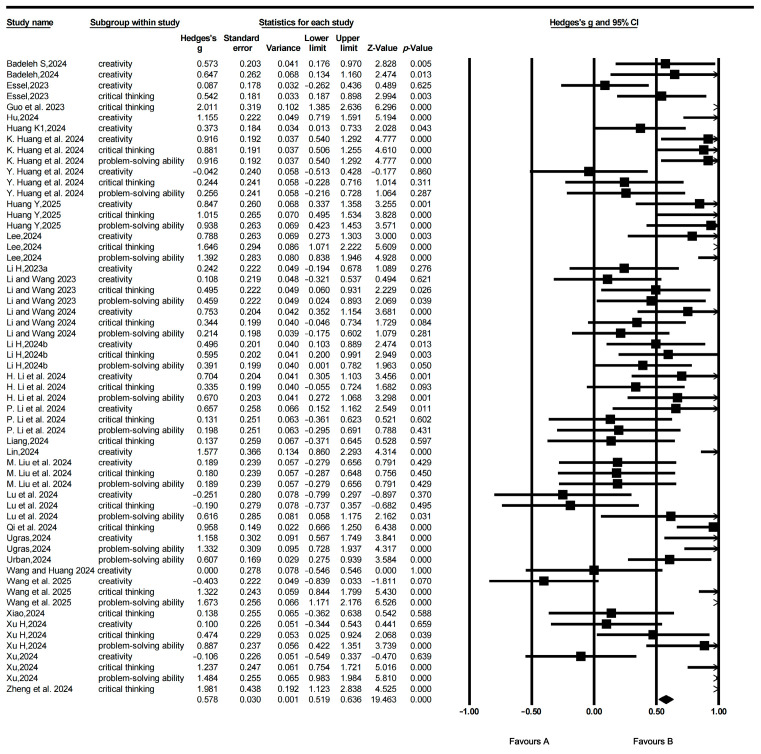
Forest plot.

Based on established methodological practices, a random-effects model was employed in this study to account for heterogeneity among the included studies. Hedges’s g was selected as the measure of effect size to assess the impact of Gen-AI on students’ HOT. As presented in Table 3, the analysis yielded an overall effect size of 0.609, indicating that Gen-AI has a moderately significant and statistically meaningful impact on students’ HOT abilities. In other words, the findings support the conclusion that Gen-AI is effective in enhancing students’ HOT skills. The effect forest plot is shown in Figure 3.

This study further examined the effects of Gen-AI on the three core dimensions of students’ HOT: creativity, critical thinking, and problem-solving, as presented in Table 4. The findings indicate that Gen-AI had the most pronounced impact on students’ problem-solving ability, with an effect size (ES) of 0.745, which falls within the moderately significant range (0.5 ≤ ES < 0.8) and is statistically significant (*p* < 0.001). This suggests that Gen-AI exerts a substantial positive influence on enhancing students’ problem-solving skills.

The second highest effect was observed in the domain of critical thinking, with an effect size of 0.691, which is also statistically significant (*p* < 0.001). This result highlights the constructive role of Gen-AI in fostering students’ critical thinking abilities.

Lastly, the effect size for creativity was 0.444, which, while lower than the other two dimensions, still represents a moderate and statistically significant effect (*p* < 0.001). This indicates that Gen-AI contributes positively to the development of students’ creative thinking, albeit to a lesser extent compared to problem-solving and critical thinking.

Taken together, these results demonstrate that Gen-AI exerts significant positive effects across all three core dimensions of HOT, with the most substantial impact observed on problem-solving, followed by critical thinking, and a moderate yet meaningful impact on creativity.

### 4.2. The Analysis of Moderator Effect Size

This study further examined the moderating effects of four potential moderator variables—intervention duration, education level, instructional method, and self-regulated learning (SRL) ability—on the relationship between Gen-AI and students’ HOT. The results are summarized in Table 5.

#### 4.2.1. Intervention Duration

When intervention duration was examined as a moderating variable, a significant between-groups difference was observed (Q = 9.106, *p* = 0.011), indicating that the length of intervention significantly moderated the effectiveness of Gen-AI on students’ HOT. Subgroup analyses revealed that interventions lasting 0–8 weeks yielded a moderate effect (ES = 0.494, *p* < 0.001), interventions of 8–16 weeks produced the most significant effect (ES = 0.759, *p* < 0.001), and interventions exceeding 16 weeks showed a comparatively more minor effect (ES = 0.372, *p* < 0.001). These results demonstrate that the impact of Gen-AI on HOT varies substantially across different intervention durations, with the 8–16 weeks window generating the most pronounced benefits.

#### 4.2.2. Educational Level

When educational level as a moderating variable indicated that the between-groups difference did not reach statistical significance (Q = 3.353, *p* = 0.067), suggesting that educational level did not constitute a statistically significant moderator in the relationship between Gen-AI and students’ HOT. Nevertheless, examination of the subgroup effect sizes revealed practically meaningful differences between K-12 and higher education contexts. Specifically, K-12 reflecting a highly significant large effect (ES = 0.857, *p* < 0.001), whereas higher education corresponding to a moderate effect (ES = 0.593, *p* < 0.001). These results imply potential differences in responsiveness or instructional context that merit further investigation.

#### 4.2.3. Instructional Method

The meta-analysis revealed that instructional methods did not exert a statistically significant moderating effect at the between-groups level (Q = 2.918, *p* = 0.232). This indicates that, overall, the moderating role of pedagogy did not reach statistical significance. Nevertheless, subgroup analyses uncovered meaningful heterogeneity across different instructional strategies. Specifically, project-based learning yielded a moderately significant effect (ES = 0.717, *p* < 0.001), blended learning produced a moderate effect (ES = 0.525, *p* < 0.001), while lecture-based instruction yielded a small, non-significant effect (ES = 0.396). These results suggest that in traditional lecture-centered contexts, the potential of Gen-AI to foster students’ HOT remains comparatively constrained.

#### 4.2.4. Self-Regulated Learning Ability

The final analysis revealed that SRL exerted a significant moderating effect on the relationship between Gen-AI and students’ HOT (Q = 40.962, *p* < 0.001). Subsequent subgroup analyses indicated marked differences in the effects of Gen-AI interventions across varying levels of SRL. Specifically, students with high SRL demonstrated a substantial positive effect (ES = 0.863, *p* < 0.001), whereas those with low SRL exhibited only a small to moderate effect (ES = 0.284, *p* < 0.001). These findings suggest that SRL constitutes a critical boundary condition shaping the educational impact of Gen-AI. The facilitative role of Gen-AI is particularly pronounced for students with strong SRL capacities, while its benefits appear comparatively limited for students with weaker SRL skills.

## 5. Discussion

### 5.1. The Effectiveness of Gen-AI on Students’ HOT

The results of this meta-analysis indicate that Gen-AI exerts a significant positive effect on students’ HOTS. The overall effect size (Hedges’s g = 0.609) represents a moderately large and statistically significant improvement, suggesting that Gen-AI plays a substantive role in supporting the development of HOTS and can serve as an effective tool for fostering students’ higher-order cognitive abilities. These findings are consistent with previous studies (Lee et al. 2024; Li et al. 2024b; Huang et al. 2024b; Ilgun Dibek et al. 2024), which collectively demonstrate that Gen-AI can function as a cognitive scaffold, provide guided prompts rather than direct answers, and create a learning environment conducive to the cultivation of HOTS. Specifically, Lee et al. (2024) developed a guidance-based ChatGPT-assisted learning tool that directs students through prompts to enhance reflective engagement, while Li and Huang further showed that teacher-mediated discussion and reflection on AI-generated feedback significantly support students’ HOT. In addition, Lu et al., using survey data from university students and structural equation modeling, found that both Gen-AI usage and evaluation directly influenced HOT, and also indirectly facilitated HOT development through behavioral engagement and peer interaction. Overall, these empirical results align with existing theoretical perspectives suggesting that technology-enhanced learning environments can provide dynamic, adaptive, and context-sensitive resources to scaffold complex cognitive processes (Bower and Vlachopoulos 2018).

At the same time, some studies have raised concerns regarding students’ overreliance on Gen-AI. Iskender (2023) and Johinke et al. (2023) reported that in writing courses, students may rely on AI not only for language refinement but also for idea generation, which could potentially undermine the development of HOT. Writing inherently integrates creative and critical processes across stages such as idea generation, argumentation, revision, and reflection; bypassing these essential processes reduces opportunities for independent reasoning and evaluative judgment. Moreover, the formulaic and homogenizing tendencies of AI-generated content may encourage “shortcut learning,” limiting originality and divergent thinking. These findings highlight the importance of discipline-sensitive instructional design: although Gen-AI holds substantial potential for enhancing HOTS, its pedagogical application must be carefully balanced. Instructional strategies should be deliberately structured—for example, by encouraging reflection, discussion, and phased use of Gen-AI—to maximize its benefits for HOT while minimizing potential adverse effects.

The analysis further revealed differential effects across the three core dimensions of HOT: problem-solving, critical thinking, and creativity. Gen-AI demonstrated the most substantial impact on problem-solving ability and critical thinking, while its effect on creativity, although positive, was relatively lower. These findings align with those reported by Lee et al. (2024), Huang et al. (2024b), and Xu et al. (2024). Specifically, Gen-AI can serve as a cognitive scaffold that guides learners through a structured process of problem identification, analysis, and resolution during interaction (Li and Wang 2023). Moreover, the real-time feedback and intelligent recommendation mechanisms embedded in Gen-AI tools can enhance learners’ metacognitive engagement during problem-solving, reduce cognitive interruptions, and promote deeper cognitive processing (Li and Wang 2024).

At the same time, the inherent limitations of Gen-AI, such as AI hallucinations, may catalyze critical thinking. The generation of inaccurate or misleading information compels students to scrutinize the validity and reliability of the outputs, thereby reinforcing their critical evaluation skills, reducing their reliance on Gen-AI, and increasing the likelihood of meaningful interactions with Gen-AI (Qi et al. 2024; Hou et al. 2025).

Although the short-term impact of Gen-AI on creativity appears to be relatively limited, its long-term potential may be significantly enhanced through systematic and intentional instructional design. First, educators can embed Gen-AI into extended, scaffolded creative tasks, encouraging students to use the tool across different stages of ideation, revision, and refinement. Such a progressive task structure provides the temporal and cognitive space necessary for iterative practice, gradually fostering creative competencies. Second, the trial-and-error process is essential for creative development. Creativity thrives in supportive, low-risk learning environments where students feel empowered to explore, experiment, and even fail. Gen-AI can facilitate this process by offering simulated environments, generating alternative ideas, or conducting data-based analysis, thus enabling students to innovate and test hypotheses with minimal risk. Through iterative experimentation and reflection, students can accumulate experiential knowledge and explore optimal creative solutions, leading to sustained development in creativity. Furthermore, Gen-AI does not directly enhance learners’ creativity but does so through mediating factors such as collective efficacy, making a collaborative environment built on mutual trust essential (Luo et al. 2025).

### 5.2. The Moderating Effects of Gen-AI on Students’ HOT

#### 5.2.1. Intervention Duration

The results indicate that the application of Gen-AI produced moderate effect sizes on students’ HOT when the intervention period lasted 1–8 weeks and 8–16 weeks, with the latter yielding more pronounced effects. This pattern appears to be closely related to students’ acceptance of Gen-AI and the psychological adaptation processes they undergo over time.

In shorter interventions (1–8 weeks), students may not have fully adapted to or mastered the use of Gen-AI as a novel educational support tool, limiting its effectiveness in facilitating HOT skills (Ganesh et al. 2022). The abbreviated exposure also restricts opportunities for in-depth exploration of the technology’s potential, thereby weakening its impact on cognitive development.

In contrast, interventions exceeding 16 weeks may lead to diminishing returns, as prolonged exposure to Gen-AI tends to foster over-reliance among students. This dependence manifests in passive acceptance of AI-generated content without critical examination and a preference for efficiency over deep, self-driven inquiry when tackling complex tasks (Du et al. 2025). Consequently, learners’ intrinsic motivation gradually erodes, diminishing their sense of autonomy and sustained engagement in the cognitive process (Wang et al. 2025). These adverse effects can attenuate the initial cognitive benefits, ultimately impeding the development of HOT.

An inverted U-shaped relationship emerges between intervention duration and effectiveness: both short-term (1–8 weeks) and long-term (over 16 weeks) interventions yield relatively limited effects, whereas the 8–16-week intermediate duration demonstrates optimal outcomes. This pattern suggests that the mid-length intervention strikes a balance, allowing students adequate time to build proficiency in using Gen-AI while avoiding the negative consequences of extended use.

To mitigate the risks associated with long-term AI integration, some pedagogical strategies are recommended. First, a phased scaffolding should be adopted, whereby AI support is systematically faded to encourage the gradual development of independent problem-solving skills (Wang et al. 2025). Second, guided Gen-AI interaction should be implemented, using carefully designed prompts to steer AI toward providing conceptual frameworks or reasoning processes rather than direct answers, thereby fostering sustained active participation and deeper cognitive engagement (Lee et al. 2024).

#### 5.2.2. Education Level

The results indicate that the educational stage does not serve as a significant moderating variable; however, the impact of Gen-AI varies across different educational stages. The analysis reveals that Gen-AI exerts a stronger influence on K-12 students than on university students. This discrepancy may stem from differences in usage patterns and instructional guidance across education levels.

In higher education, students tend to rely on Gen-AI primarily for text generation and information retrieval, often engaging in superficial interactions or delegating intellectual tasks to the technology—a phenomenon referred to as “knowledge outsourcing” (Li et al. 2024c). Many college students pose low-level questions to Gen-AI for tasks such as assignments, translations, and essay writing. Prior studies (Liu et al. 2024b) have shown that some students plagiarize AIGC, which hinders the development of deep learning and critical thinking. Similarly, in programming tasks, students often copy and paste faulty Gen-AI-generated code without verification, reflecting a lack of reflective engagement (Sun et al. 2024).

In contrast, K-12 students are more likely to use Gen-AI as a guided learning tool under teacher supervision. They employ Gen-AI in exploratory learning and problem-solving, suggesting that appropriate instructional scaffolding is critical to ensuring productive use. As Shi and Han (2024) have pointed out, in the absence of teacher guidance, students’ excessive reliance on AI-generated content may undermine their critical thinking skills and autonomy.

Regardless of the educational stage in which Gen-AI is applied, teachers play a pivotal role as facilitators of student learning. In classroom settings, their ability to strategically leverage Gen-AI technologies is essential for fostering HOT. Therefore, it is recommended that educators receive targeted professional development on how to integrate Gen-AI tools effectively into instructional design, ensuring that technological affordances are aligned with pedagogical goals to maximize cognitive engagement and thinking depth.

#### 5.2.3. Instructional Method

Although the overall moderating effect of the instructional method was not statistically significant, substantial differences were observed in the effect sizes across pedagogical approaches. Notably, the combination of project-based learning (PBL) with Gen-AI yielded the most positive outcomes. This aligns with findings by Shahzad et al. (2024), who demonstrated that the integration of Gen-AI significantly enhances the effectiveness of PBL and supports the development of student creativity.

PBL emphasizes learner-centered, inquiry-driven activities, where students engage in real-world projects to construct knowledge (Kimberly 2023). In this context, Gen-AI acts as a valuable cognitive partner, offering diverse resources and perspectives, and enabling students to explore and solve complex problems autonomously (Khan et al. 2023).

In contrast, blended learning outcomes depend heavily on students’ self-regulation and digital literacy. Learners with low adaptability or limited support may misuse Gen-AI, leading to unstructured engagement and reduced learning quality. This is consistent with Means et al. (2013), who noted that less adaptive learners often struggle in blended environments.

Under lecture-based instruction, students typically engage in passive learning, relying on teacher-led knowledge transmission. Gen-AI is often relegated to a peripheral role as a supplementary information source, with limited impact on students’ active engagement or cognitive transformation.

In light of these findings, educators are encouraged to expand the use of PBL and provide structured guidance on Gen-AI integration. Furthermore, educators should place greater emphasis on instructional design and the deliberate selection of pedagogical approaches. Instructional planning should be informed by a careful analysis of learner characteristics, enabling the design of targeted learning activities and provision of appropriate resources to meet students’ needs in AI-enhanced environments (Chiu et al. 2023). By scaffolding students’ engagement with Gen-AI tools for self-directed inquiry and problem-solving, educators can better leverage the affordances of Gen-AI to support the development of HOT skills.

#### 5.2.4. Self-Regulated Learning Ability

Self-regulated learning (SRL) plays a critical role in enabling students to establish learning goals, select appropriate strategies, and monitor their cognitive processes, thereby laying a foundational framework for the development of HOT. As emphasized by Wang and Liu (2024), students’ SRL capabilities may directly and significantly enhance their HOT competencies. Conversely, engagement in HOT tasks, such as analyzing complex problems, constructing reasoned arguments, or evaluating evidence, requires continuous goal-setting, strategic planning, and metacognitive reflection. This dynamic process, in turn, reinforces and further develops students’ self-regulatory abilities.

This study, through empirical analysis, confirms that SRL exerts a significant moderating effect on the relationship between Gen-AI and HOT skills. This finding resonates with the results of Xu et al. (2024), Rusandi et al. (2023), and Wang and Huang (2024), further reinforcing the notion that SRL, as a critical individual difference variable, plays a central role in determining whether external intelligent tools can be effectively leveraged to promote learners’ cognitive development (Xu et al. 2024; Rusandi et al. 2023). Specifically, Wang and Huang (2024) emphasizes that SRL functions as a “key regulatory mechanism” that can effectively “neutralize” the potential cognitive risks associated with the use of Gen-AI in fostering HOT.

The results indicate that students with higher levels of SRL are capable of proactively planning their learning trajectories rather than passively relying on or directly replicating Gen-AI–generated outputs. Such students tend to critically reflect on the generated content, seeking to integrate it with their existing knowledge structures rather than adopting it uncritically (Zhou et al. 2024; Borge et al. 2024). Moreover, students with high SRL demonstrate a heightened capacity to recognize the educational value of Gen-AI tools, actively incorporating them into their learning processes. By employing critical thinking and evaluative skills, they can extract valuable information, thereby enhancing problem-solving abilities and fostering creative thinking.

In contrast, students with lower levels of SRL often struggle to assess the accuracy and relevance of AI-generated content, making them more susceptible to uncritically accepting information, including errors or misleading outputs (Xu et al. 2024). They also face difficulties in aligning Gen-AI tools with their learning objectives and lack the flexibility to adapt strategies when confronted with challenges (Wang and Huang 2024). Consequently, their use of intelligent tools remains inefficient, hindering their engagement in effective knowledge construction.

Taken together, these findings underscore the pressing need for educational practice to prioritize and systematically strengthen students’ SRL capacities. By designing targeted instructional interventions and adopting tiered strategies for Gen-AI integration, educators can support the development of adaptive learning behaviors. In particular, fostering students’ metacognitive awareness, strategic planning, and reflective learning practices can enhance their ability to critically and autonomously engage with Gen-AI. Ultimately, such efforts can maximize the educational potential of Gen-AI in advancing HOT skills.

## 6. Conclusions and Limitations

**Figure 4:**
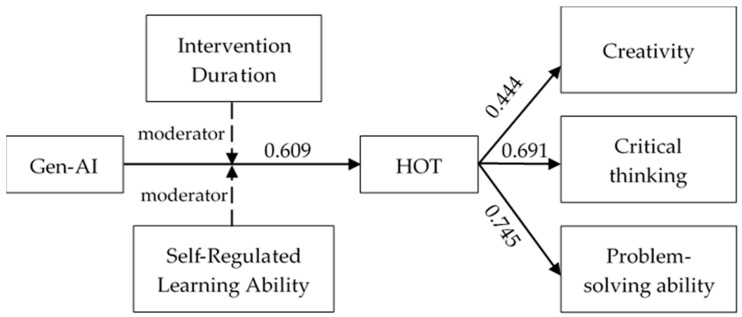
Model of findings.

The present meta-analysis reveals that Gen-AI exerts a moderate positive influence on the development of students’ HOT, with a moderately significant positive impact on problem-solving ability and critical thinking, yet a relatively more minor effect on creativity. However, its effectiveness is significantly moderated by factors such as intervention duration and students’ self-regulated learning abilities (see Figure 4). Specifically, the impact of Gen-AI is most pronounced when the intervention lasts between 8 and 16 weeks, whereas interventions shorter or longer than this duration yield comparatively weaker effects. Moreover, students with higher self-regulated learning abilities benefit more substantially from Gen-AI than those with lower self-regulation skills, underscoring the critical role of learner agency in technology-enhanced learning environments. These findings underscore the importance of tailoring Gen-AI implementation to intervention duration and learner differences, notably SRL. This study offers theoretical and practical guidance for integrating Gen-AI, highlighting its potential to foster HOT, a core component of innovation.

Despite its contributions, this study has several limitations that warrant further exploration. First, the scope and diversity of the sample and research designs limit the generalizability of the findings. The inclusion of a relatively narrow range of educational contexts and learner populations may restrict the external validity of the results. Moreover, this meta-analysis was limited to studies published in Chinese and English. Such a language restriction could introduce potential bias by omitting literature in other linguistic contexts, which in turn undermines the generalizability of the findings. Future investigations ought to encompass more diverse educational contexts and student populations to enhance the robustness and applicability of the conclusions, while employing more inclusive methodologies to cover a wider spectrum of research literature.

Second, the study may not fully capture the rapidly evolving nature of Gen-AI technologies and their expanding roles in educational practice. The current meta-analysis is based on existing literature, which may lag behind the most recent developments and emerging applications. To address this, future studies should employ longitudinal designs, cross-cultural comparisons, and multi-dimensional analytical models and incorporate broader dimensions of HOT (e.g., decision-making and evaluation) to understand better the complex and dynamic effects of Gen-AI on HOT. Such approaches will provide more nuanced and context-sensitive insights, thereby offering more targeted guidance for the integration of Gen-AI in educational practice.

## Figures and Tables

**Table 1:** Core Dimensions of Higher-Order Thinking Across Disciplines.

Scholar (Year)	Discipline	Core Dimensions of HOT
Liu (2022)	Mathematics Education	Critical thinking, creative thinking, problem-solving skills, metacognition
Xu et al. (2024)	Information Technology Education	Problem-solving ability, computational thinking, creativity
Tan and Cho (2021)	Translation Studies	Critical thinking, creative thinking, communicative thinking, affective cognition
Hwang et al. (2018)	Cross-disciplinary	Collaboration, communication, complex problem-solving, critical thinking, creativity
Alkhatib (2022); Yang (2015); Ilgun Dibek et al. (2024)	Cross-disciplinary	Critical thinking, problem-solving ability, creativity

**Table 2:** Code of moderator.

Variable	Code
Intervention Duration	1 = 0–8 weeks
	2 = 8–16 weeks
	3 = >16 weeks
Educational Stage	1 = K-12 (Kindergarten through 12th grade)
	2 = Post-secondary (undergraduate through graduate)
Instructional Method	1 = lecture-based
	2 = project-based
	3 = blended
Self-Regulated Learning Ability	1 = high SRL ability
	2 = low SRL ability

**Table 3:** Heterogeneity test results.

Model	Number Studies	Effect Size	95% Confidence Interval		Heterogeneity			
			Lower Limit	Upper Limit	Q-Value	df	*p*-Value	I-Squared
Fixed	59	0.578	0.519	0.636	255.208	58	0.000	77.273%
Random	59	0.609	0.485	0.732				

**Table 4:** Test results for the effect of Gen-AI on three dimensions of HOT.

Higher-Order Thinking	N	ES	95%CI		Two-Tailed Test		Heterogeneity		
			Lower Limit	Upper Limit	Z-Value	*p*-Value	Q-Value	df	*p*-Value
Creativity	23	0.444	0.259	0.629	4.698	<0.001	4.961	2	0.084
Critical thinking	20	0.691	0.464	0.918	5.973	<0.001			
Problem-solving ability	16	0.745	0.521	0.970	6.507	<0.001			

**Table 5:** Tests of the moderating effect.

Moderator	Subgroup	N	ES	95%CI		Two-Tailed Test		Intergroup Effect
				Lower Limit	Upper Limit	Z-Value	*p*-Value	
Intervention Duration	0–8 weeks (1)	15	0.494	0.272	0.717	4.350	<0.001	Q = 9.106 *p* = 0.011
	8–16 weeks (2)	31	0.759	0.576	0.943	8.113	<0.001	
	>16 weeks (3)	13	0.372	0.196	0.549	4.133	<0.001	
Educational Stage	K-12 (1)	13	0.857	0.542	1.172	5.335	<0.001	Q = 3.353 *p* = 0.067
	Post-secondary (2)	46	0.539	0.412	0.667	8.263	<0.001	
Instructional Method	lecture-based (1)	6	0.396	−0.088	0.879	1.605	>0.001	Q = 2.918 *p* = 0.232
	project-based (2)	31	0.717	0.507	0.928	6.674	<0.001	
	Blended (3)	22	0.525	0.406	0.644	8.636	<0.001	
Self-Regulated Learning Ability	High (1)	31	0.863	0.679	1.048	9.181	<0.001	Q = 40.962 *p* = 0.000
	Low (2)	25	0.284	0.188	0.380	5.788	<0.001	

## Data Availability

Readers can request the data from the corresponding author.

## Abbreviations

The following abbreviations are used in this manuscript:

Gen-AI	Generative Artificial Intelligence
HOT	Higher-order Thinking
SRL	Self-regulated Learning
PBL	Project-based Learning
